# Recovery of Surgical Training Through Extended Laparoscopic Simulation Training

**DOI:** 10.7759/cureus.18695

**Published:** 2021-10-12

**Authors:** Mohammed Hamid, Zohaib Siddiqui, Shaheer Aslam Joiya

**Affiliations:** 1 General Surgery, University Hospitals Birmingham NHS Foundation Trust, Birmingham, GBR; 2 General Surgery, Maidstone and Tunbridge Wells NHS Trust, London, GBR; 3 Trauma and Orthopaedics, Yeovil District Hospital, Yeovil, GBR

**Keywords:** laparoscopic skills, training recovery, surgical residency program, simulation training, laparoscopic surgery

## Abstract

Introduction

The coronavirus disease 2019 (COVID-19) pandemic has adversely affected surgical training internationally. Laparoscopic surgery has a steep learning curve necessitating repetitive procedural practice. We evaluate the efficacy of short- and long-duration simulation training on participant skill acquisition to support the recovery of surgical training.

Methods

A prospective, observational study involving 18 novice medical students enrolled in a five-week course. Nodal timed assessments involved three tasks: hoop placement, stacking of sugar cubes and surgical cutting. One month post-completion, we compared the ability of six novice course participants to that of six surgical trainees who completed a smaller portion of the course curriculum.

Results

Course participants (n=18) completed tasks 111% faster on their third and last course attempt. The surgical trainee group (n=6) took 46% longer to complete tasks compared to the six re-invited course participants, whose ability continued to advance on their fourth effort with a combined 154% earlier completion time compared to try one.

Conclusions

This study supports the adoption of a structured, extended, regular and spaced-out simulation course or curriculum to cultivate greater skill acquisition and retention amongst surgical trainees, and improve patient care.

## Introduction

Surgical training in the United Kingdom (UK) and internationally has taken a tremendous toll in the face of the severe acute respiratory syndrome coronavirus 2 (SARS-CoV-2) pandemic [1-4]. UK trainee logbooks displayed a 50% reduction in trainees as the primary surgeon, with widespread apprehension amongst all residents on meeting annual competencies [5,6]. Trainees have used the hashtag #NoTrainingTodayNoSurgeonsTomorrow to voice their educational concerns [7]; and a recent collaborative commitment to improve surgical training has been outlined by the Joint Committee on Surgical Training, the Association of Surgeons in Training, the British Orthopaedics Trainees’ Association and the Confederation of Postgraduate Schools of Surgery [5]. Efforts to recover training have begun, and teaching Trusts have been advised to review their training framework to re-inject effective methods and opportunities into their programs.

Laparoscopic simulation has been demonstrated in numerous studies to significantly benefit trainee skill acquisition whilst supplementing real time theatre exposure [8,9]. In the UK, specialized centres of excellence and training institutes are offering courses to complement training, however, many in-house training programs conduct very little to no laparoscopic simulation teaching or standardized simulation curriculum. Where courses are available, most tend to be short-term, spanning days rather than weeks, with no recorded evidence of how they impact the trainee in the long run.

The pandemic has also left a colossal backlog of five million elective surgical cases within the UK, of which laparoscopic procedures make up a large proportion [10]. This raises the question as to whether incorporating an internal laparoscopic simulation curriculum would enhance training and tackle the excess workload. This study aimed to determine the objective and subjective value of five weeks of laparoscopic simulation training and practice. We also compare the ability of the novice trainees (medical students and foundation year doctors, interested in a surgical career) who participated in the course, against a cohort of invited core surgical trainees (CST) using the same standardized simulation exercises.

This article was previously presented as a meeting abstract at the ASiT x MedAll International Surgical Summit on October 17, 2020.

## Materials and methods

This is a prospective, observational study involving 18 foundation (first post-graduate) year doctors and fifth-year medical students lacking prior laparoscopic exposure, from London, England. All participants were recruited on a volunteer basis and completed a written informed consent document prior to starting. Candidates were enrolled onto a structured five-week simulation course designed by trainees under the guidance of two Consultant General Surgeons. Participants had an introductory lecture to the course explaining the ensuing five two-hour sessions, and were provided with a complimentary booklet outlining the set of skills to be learned and practiced.

Feedback on the session schedule was requested throughout the course to maintain 100% retention rate. The facilitator’s objective was to educate the candidates on technique refinement. Three timed and standardized tasks were undertaken and recorded at the beginning, middle and end of the course to inspect for time improvements.

**Figure 1:**
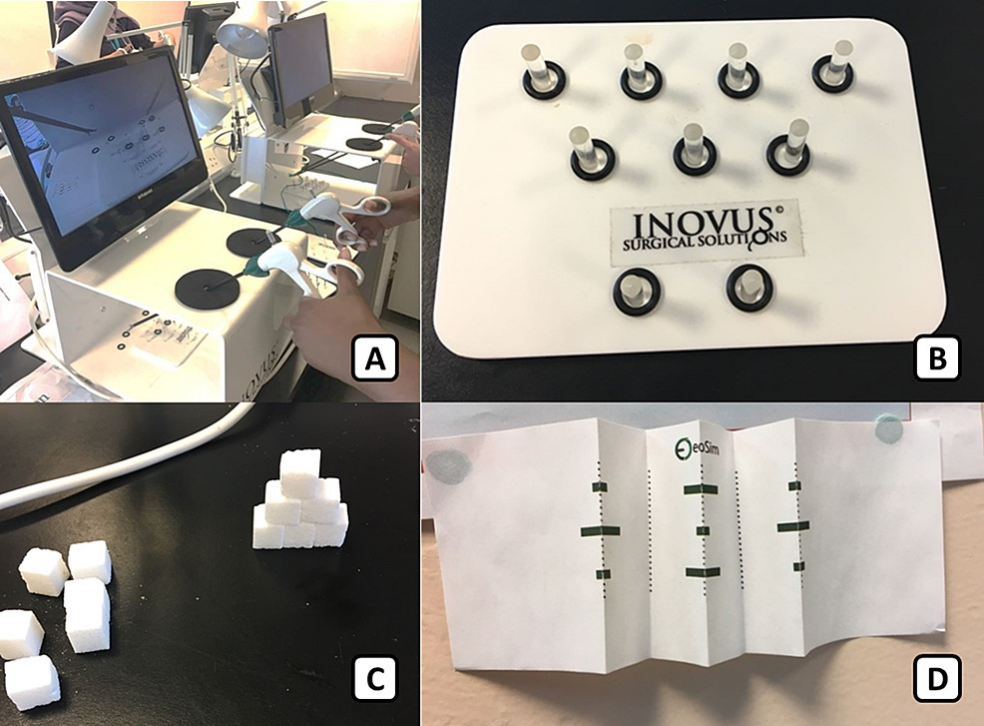
Demonstrates the three tasks completed by each candidate on the Inovus laparoscopic simulation box-trainer (1A). (B) Hoop placement: The participants were required to use the laparoscopic graspers to place nine plastic rings from a single file onto nine separate plastic pegs. (C) Stacking sugar cubes: The participants were required to use the laparoscopic graspers to stack the sugar cubes into a pyramid with three cubes at the base. (D) Surgical cutting: The participants were required to use the laparoscopic scissors to cut across nine black lines on a folded paper. To regulate the level of precision, participants forfeited a five second penalty for each cut beyond the lines.

The laparoscopic equipment used was Inovus Medical (St Helens, UK) laparoscopic box trainers [11], located within the clinical skills lab (Figure 1A). The three timed tasks were hoop placement, stacking of sugar cubes and surgical cutting (Figure 1B-1D). These tasks were chosen to study each individual’s development of spatial awareness, fine dexterity and accuracy. These exercises were also felt to be representative of the basic manual skills required in many laparoscopic tasks, illustrating a transferable skill-set.

Four weeks after the course had ended, we invited back six of the “novice” course participants on a volunteer basis, and six other volunteer core surgical trainees (first-year surgical residents) with logbook experience in a range of laparoscopic and open surgical training: from handling the camera, to completing laparoscopic appendectomies and assisting in laparotomies. Both groups undertook the equivalent of one course session before completing the same three timed tasks. The recoded results allowed for objective comparison of a novice cohort who partook in a “long-term” course, against a relatively more clinically experienced group who undertook a lesser portion of the same standardised simulation training. We also scrutinized any further improvements in the aptitude of the novice group, i.e. their fourth attempt.

## Results

During the five-week course, we observed time improvements in all three tasks for all the novice participants (n=18). Across this cohort, we witnessed a mean 91% faster completion time for hoop placement, sooner by 133% for sugar cube stacking, and 108% for cutting straight lines. Participants more than halved (111%) their completion time across all tasks by their third attempt, which marked the end of the training.

**Figure 2:**
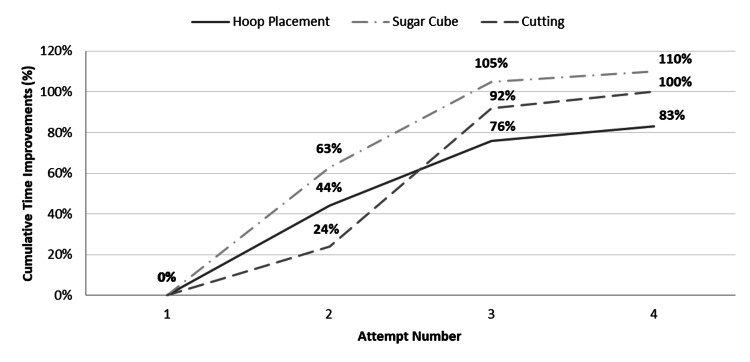
Demonstrates the percentage improvements in completion times made between each attempt for the three tasks. Attempt 1-3 (n=18, five-week course participants), attempt 4 (n=6, re-invited novice course participants cohort one month later).

On their fourth attempt, one month later, the re-invited novice group (n=6) demonstrated continued improvements, with a combined 154% earlier completion time compared to their first attempt, signalling retention and ongoing internal refinement of the learned motor skills. Specifically, hoop placement was completed 7% sooner than their third attempt, earlier by 5% for sugar cube stacking, and by 8% for cutting straight lines. Figure 2 illustrates a plateau in the cumulative time improvements between each consecutive attempt across the novice cohort.

**Figure 3:**
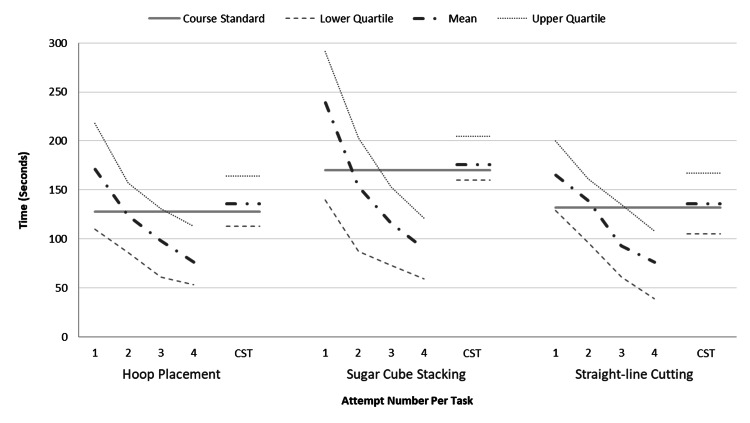
The “course standard” line demonstrates the mean time taken to complete each task across all course attempts (n=18). The mean, lower and upper quartile lines are presented for each attempt, task and group. Attempt four was undertaken by a group of six course participants one month later. CST indicates the invited core surgical trainees (n=6) first attempt one month after the course.

A “course standard” time was calculated for each of the three tasks using the mean of the entire course attempts to generate the benchmark duration for completing each task. The task standards were used to objectively compare against the attempts on the course and that of the re-invited novice and CST groups. Figure 3 represents the mean and interquartile times for each attempt and task for all groups.

The mean time taken to complete the tasks on the third attempt (n=18) of the course was 40% faster than the course standard, and 77% faster on the fourth attempt when undertaken by the six course participants one month later. In comparison, the CST group was 4% slower than the course standard times and took longer than the third and fourth novice attempts by 31% and 46% respectively, demonstrating the importance of prolonged spaced-out repetition and the specificity of these tasks (Table 1). The core surgical trainees did perform better than the novices’ first try indicating the use of transferrable skills from theatre to simulation.

**Table 1:** Demonstrates the calculated course standard time for each task in seconds (s); the mean time (s) and percentage improvement for the third course attempt (n=18), the fourth novice attempt (n=6), and the first CST attempt (n=6). CST: Core Surgical Trainee Cohort

Tasks	Course standard (*s*)	Course 3^rd ^Attempt (*s*, %)	Novice 4^th ^Attempt (*s*, %)	CST Attempt (*s*, %)
Hoop Placement	128	98 (31%)	76 (68%)	136 (-6%)
Sugar cube stacking	170	116 (47%)	90 (89%)	176 (-3%)
Straight-line cutting	132	93 (42%)	76 (74%)	136 (-3%)

**Figure 4:**
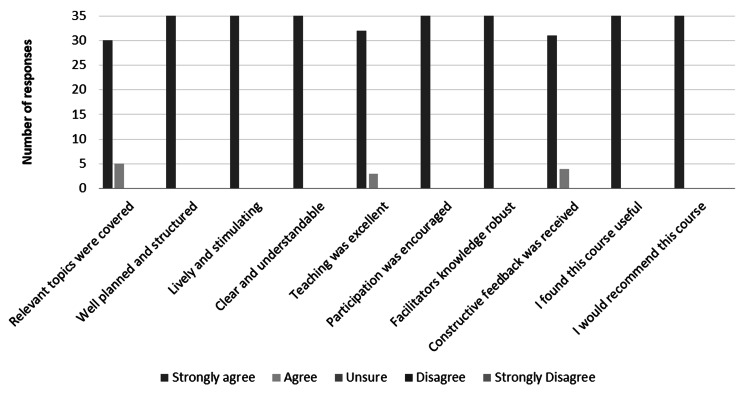
Five-week course feedback form results; 97% response rate (n=36).

We provided the 18 course participants with the same feedback form both at the beginning and the end of the course to understand how this experience impacted their training, and to provide quality improvement changes. We recorded a 97% response rate (n=36). All partakers agreed or strongly agreed that the course was useful, relevant, well planned and would be beneficial for their peers, as shown in Figure 4. In their additional comments, participants admitted that the course was inspirational and “encouraged [them] to consider pursuing surgery as a possible career choice”.

## Discussion

The veracity of laparoscopic surgery was questioned during its inception [12]; however, minimally invasive surgery continues to replace standard open surgical procedures with consistent, culminating evidence for improved postoperative outcomes [13,14]. In 2018, an estimated fifteen million laparoscopic procedures were being performed globally, with an industry worth $18.39 billion [15].

Attainment of laparoscopic skills presents surgeons with technical challenges previously not encountered in open-surgical training. Reduced tactile sensation, poor depth perception, a two-dimensional visual field, confined working spaces, and the counterintuitive manipulation of instruments are just some of the practical factors prolonging the learning curve [16].

In laparoscopy, the learning curve follows the same general pattern observed with acquiring many other motor skills: the majority of skill acquisition takes place at the beginning, with additional minor improvements and refinements ensuing from continued practice [17]. This study witnessed a fall in time improvements with each attempt as the participants approached their “optimal” task time, possibly representing a learning curve (Figure 2). Towards the end of the course the disparity in time between all applicants narrowed significantly and the degree of variability more than halved across all tasks performed. However, the observed learning curves did not plateau horizontally after the five-week course, indicating that longer practice was required for mastery.

Expertise in laparoscopic surgery is acquired through a program of detailed training and continuous practice [18,19]. The educational psychologist Benjamin Bloom explained the psychomotor process involved in learning: with constant repetition, the time taken to complete a task decreases as the motor synapses are refined in the cerebellum [20]. Increased practice and exposure have also been correlated with significantly lower complication rates [21-23].

Studies in the literature also support a spaced-out approach to learning laparoscopic skills, as it takes several spaced-out attempts to achieve the initial steep learning phase [24-26]. Within this study, the re-invited novice group continued to show time improvements on their fourth attempt one month later, though we noticed a steeper drop in their time improvements, demonstrating that a more regularly distributed training program is ideal.

Simulation training has been shown on multiple occasions to help improve laparoscopic skills, time efficiency, as well as tackle complication rates [8,9,27]. Simulators can provide objective feedback such as task-time, evaluation of movement patterns, and allow for a supervisor to objectively evaluate the trainee’s laparoscopic skills which can often be difficult in the operating room [28]. The use of simulation training as an adjunct to live training provides a safe space for the trainee to continue their development in laparoscopic skills. However, it can be expensive and needs the relevant amount of resources, space and time.

Surgeons and trainees are aware of the need for development of the surgical training program, and this has given rise to the Improving Surgical Training (IST) pilot [29]. Most laparoscopic procedures have a long learning curve which means that residents need to repetitively practice the operations. Short-term condensed courses introduce fatigue, give the cerebellum less time to refine the motor synapses, and are associated with a reduced retention rate [24-26]. A standardised, systematic in-house simulation curriculum for acquiring laparoscopic skills, along with continuous practice and sufficient exposure under trained specialists, will aid training recovery [30].

Limitations

There are several limitations to this study, primarily the size of the cohorts analysed limited by the resources and volunteers available. Randomisation of the re-invited novice group was not possible due to participant availability. The timed-task attempts were strictly completed once and do not represent the best time out of multiple attempts. Though the later may introduce chance error, surgeons are limited to one attempt whilst operating in-vivo. This study does not factor in other key components of the laparoscopic learning curve such as economy of movement, procedural safety, the transferability to live theatre, or complication rates.

The learning curves plotted within this research do not plateau despite the study spanning over two months. Though this study is unable to specify the optimal training length requisite for mastery, it implies a longer duration than that provided by short-term courses.

This study does not compare short- and long-duration simulation training utilising groups with similar laparoscopic experience. The CST group had considerable prior in-vivo practice and undertook the equivalent of one course session denoting a short training period. Despite the background of the CST group, they were outperformed by the novice group who undertook a longer duration of training, strengthening our hypothesis for longer-term simulation training courses and curriculums.

## Conclusions

The results of this study support the proposition for structured, extended, regular and spaced-out simulation courses or curriculums to advance laparoscopic skill acquisition within resident training. Repetition in simulation, with nodal point assessments, can further reinforce trainee experiences in-vivo and favours patient outcomes. This study encourages a more in-depth investigation into an in-house integrated simulation curriculum to assist in training recovery, tackle the projected workload as a consequence of the COVID-19 pandemic, and stimulate medical students interested in a surgical career.
